# Thermal Energy Storage Using a Hybrid Composite Based on Technical-Grade Paraffin-AP25 Wax as a Phase Change Material

**DOI:** 10.3390/nano13192635

**Published:** 2023-09-25

**Authors:** Hossam A. Nabwey, Maha A. Tony

**Affiliations:** 1 Department of Mathematics, College of Science and Humanities in Al-Kharj, Prince Sattam bin Abdulaziz University, Al-Kharj 11942, Saudi Arabia; 2 Basic Engineering Science Department, Faculty of Engineering, Menoufia University, Shebin El-Kom 32511, Egypt; dr.maha.tony@gmail.com; 3 Advanced Materials/Solar Energy and Environmental Sustainability (AMSEES) Laboratory, Faculty of Engineering, Menoufia University, Shebin El-Kom 32511, Egypt

**Keywords:** thermal energy storage (TES), phase change materials (PCM), composite, water heating

## Abstract

Thermal energy storage (TES) has a strong ability to store energy and has attracted interest for thermal applications such as hot water storage. TES is the key to overcoming the mismatch between energy supply and demand by using phase change materials (PCMs). However, a common organic PCM characteristic is low thermal conductivity. This causes a slow thermal response for paraffin-AP25, which is not suitable for many applications. Hence, a search is underway for modifications to enhance its thermal properties. Thus, the current investigation introduces a novel PCM system based on the use of waste material as an economic and efficient system. In the current investigation, nanoparticles were added to a PCM; specifically, a technical-grade paraffin-AP25 wax (AP25 wax)/hybrid composite was synthesized via ultrasonic dispersion. The focus of this investigation is to assess the behavior of a PCM for energy storage via charging (melting process) and discharging (solidification process). Nanoparticles of magnetite were prepared via a simple, cost-efficient route, co-precipitation, augmented with aluminum and silicon derived from waste streams from a hydrothermal facility and mixed with paraffin-AP25 wax to form a hybrid composite PCM. Transmission electron microscopy and scanning electron microscopy (augmented with dispersive X-ray analysis, EDX) micrographs, in addition to X-ray diffraction (XRD), show the prepared composite. Different mass fractions of the composite, ranging from 1 to 10 weight %, were embedded in a paraffin-AP25 phase change material. The latent heat storage capacity of the PCM was enhanced by 8% when paraffin-AP25 was applied. Finally, the overall system efficiency was evaluated, and the yield increased by 64% for the 8% hybrid composite.

## 1. Introduction

Worldwide, there has been an increase in energy needs due to changes in lifestyle in terms of modern industry and domestic life [1]. As such, there is an increase in the consumption of conventional fossil fuel sources and, thus, an increase in the emissions of carbon dioxide. According to the statistics of the IEA (International Energy Agency), there has been an approximately 20% increase in energy consumption in recent decades. Such challenges motivate industrial sectors and scientific researchers to investigate practical solutions to overcome these environmental issues and create a sustainable energy alternative.

The physical state of phase change materials can be changed, and they are proficient in storing TE by releasing latent heat. Such systems can occur via cyclic melting and freezing processes. New trends suggest PCMs as a promising option, using distinct solid-phase composite materials as a key technology. Such composites may be in the amorphous phase or, more often, in the crystalline phase [2]. The stored energy can be used for numerous applications, including domestic building needs such as water heating and district heating, as well as industrial essentials, such as in the food and chemical industries [3] and in drying [4]. Additionally, in their advanced form, PCMs can be introduced into thermodynamic solar plants [4]. Enhancing the thermal conductivity of PCMs can efficiently maximize system efficacy [5,6]. Therefore, research on PCM systems as TES media has increased.

The economic growth and development of commercial-grade PCMs is limited due to their material characteristics. Although latent heat energy storage (LHS) possesses the most flexible operating temperature ranges, it is not as widely applied as sensible heat energy storage [7]. Many experimental studies have been carried out using various media in heat storage systems [8,9]. To date, water is widely used as a sensible heat storage material. However, the disadvantages associated with its use are linked to its low storage density and high insulation [9]. The advantages associated with sensible heat energy storage over sensible heat are associated with the fact that it can reach up to 10-fold higher stored energy than sensible heat [6]. The high thermal conductivity of LHS and its superior heat of fusion compared to those of sensible heat are promising for such applications [10].

Recently, novel substances have been introduced to design such systems [11,12]. The application of PCMs involves solid-to-liquid phase transition and vice versa in a round-cycle system [12]. Presently, numerous synthetic or natural types of PCMs have been introduced into energy storage systems. PCMs can be classified as organic [13], inorganic [14], or eutectic [15]. Eutectic phase change materials, with their exceptional characteristics, are categorized as multi-substances, as they avoid separation or supercooling criteria. However, from a commercial point of view, the cost of eutectic phase change materials remains a main problem associated with their applications. However, organic phase change materials are characterized by self-nucleation, which means crystallization with or without minimal supercooling. Further, they should be non-corrosive in nature. Nevertheless, their low latent heat is still an obstacle to their applications [13]. Further, inorganic materials such as salt hydrates have a low temperature range, and some salt hydrates are toxic and corrosive in nature [14]. Thus, a search is underway for a combination that possesses new advantages.

In this regard, numerous types of PCMs can be used for TESs [13] which might be well insulated for less sophisticated and cost-efficient systems, with special attention to paraffin materials [14,15]. Such paraffin-based substances possess a large amount of latent heat energy but negligible super-cooling and have a suitable melting temperature profile [16]. However, the merits of nanoparticles or inorganic materials embedded in PCMs can resolve these issues due to their effective thermophysical properties [17]. For instance, Bianco et al. [17] used a micro-encapsulated phase change material integrated into a commercial water tank for cold thermal energy storage improvement. Nematpour Keshteli et al. [18] embedded 5 weight % nanoparticles in pure paraffin wax and improved its PCM performance in the melting cycle, improving the overall efficiency. Sharma et al. [19] incorporated carbon nanotubes into paraffin to enhance PCM performance. Thus, the unique physical and chemical characteristics associated with such materials are responsible for enhancing PCMs [20]. Such materials can maintain extra latent heat storage capacity [20]. Hence, future research is required to enhance such embedded PCM systems.

This article aims to improve the thermal conductivity of paraffin-AP25 by adding economic materials from waste streams and highly magnetic and conductive Fe_3_O_4_. Such materials are used in hybrid composites to increase the thermal conductivity of paraffin-based PCMs to introduce properties appropriate for PCM applications and overcome cost restrictions. The paraffin-AP25composite was prepared via the dispersion technique at different weight fractions to enhance the thermal properties of paraffin-AP25. The targeted applications ofparaffin-AP25 composites include systems that require efficient and fast heat storage and recovery facilities. Thus, the current investigation introduces a novel PCM system based on the use of waste material as an economical and efficient system.

## 2. Materials and Methods

### 2.1. Materials

A block of technical-grade paraffin-AP25 wax (AramisCo. Dallas, Texas, USA) with a melting point of about 48–53 °C and a latent heat of fusion of 190 KJ/kg was used in the current study. A slight difference is recorded between technical-grade paraffin-AP25 and commercial paraffin, with a 2 °C difference in melting temperature between the two materials [7]. Paraffin-AP25 wax has a linear, cyclic, or branched structure (C_n_H_2n+2_) [21]. The main thermophysical characterization of paraffin-AP25 wax at room temperature is given in Table 1 [7].

### 2.2. Paraffin-AP25/Composite PCM Preparation

Aluminum-based waste sludge residuals, as a by-product of waterworks, were collected from a water treatment plant. The collected slurry waste was subjected to gravity settling followed by air-drying. Afterward, the dried sludge cake was incinerated at 400 °C (2 h) in an electrical furnace and ground in a ball mill for 1 h to obtain a fine powder according to the previously reported method [21]. Then, 15g of paraffin-AP25 was mixed with the composite and dispersed via ultrasonication at 60 °C in an ultrasonic bath. The composite was mixed in the content range of 0 to 10%. Initially, the paraffin-AP25 was melted at 62 °C; afterward, the desired amount of composite was added. The mixture was exposed to strong ultrasonication for 30 min at 40 kHz at 60 °C using an ultrasonic bath (DAIHAN Wisd model WUC-A03H, 40KHz) in order to attain good dispersion of the hybrid composite within the paraffin wax PCM. The melted composite PCM was then ready for use in the heat exchanger.

### 2.3. Experimental Methodology

**Figure 1:**
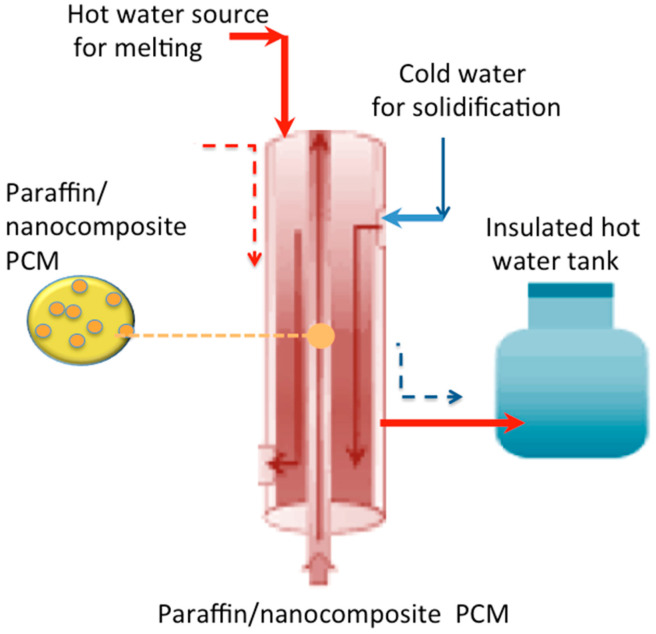
Schematic overview of the process.

Initially, the melted PCM after sonication was inserted into the tube inlet of the exchanger. Water was used as the heating fluid and was passed through the shell of the shell-and-tube heat exchanger for the cyclic melting and solidification cycles (charging/discharging) of the paraffin-AP25 PCM and hybrid PCM inside the tube. The cooling cycle was contingent on the water flowing in the shell of the heat exchanger. To avoid heat losses, the hot water storage tank was well insulated and was used for collecting hot water from the discharging cycle. All the parts were connected in parallel via a rubber tube piping system, with fluid flow at a definite flow rate, and the system was installed indoors in the laboratory. After the discharging cycle, the heat exchanger was heated in order to remove the PCM and prepare the system for the next run. A schematic overview of the process is illustrated in Figure 1.

### 2.4. Experimental Measurements and Characterization

In order to determine the temperature at different points, digital thermocouples were used and mounted in various places. Such thermocouples were mounted on the inlet and outlet of the heat exchanger to investigate the system efficiency. One thermocouple was inserted into the paraffin-AP25/composite PCM to measure the heat stored.

X-ray diffraction (XRD) was measured using an XRPhillipsX’pert (MPD3040) X-ray diffractometer supported by a monochromatic Cu Ka source (k = 1.5406°A) to characterize the prepared composite. The measurement was conducted in the step-scan mode and taken every 0.02° in the range from 20 to 80°. Transmission electron microscopy was conducted using a JEM-2100 instrument to investigate the morphology of the composite (type Tecnai G20, FEI). Further, samples were explored and imaged using a field-emission scanning electron microscope (SEM) (FE-SEM, Quanta FEG 250), and the main elements contained in the images of catalyst samples were assessed via examination of the energy-dispersive spectrum (EDX).

## 3. Results and Discussion

### 3.1. Characterization of the Composite

#### 3.1.1. XRD

**Figure 2:**
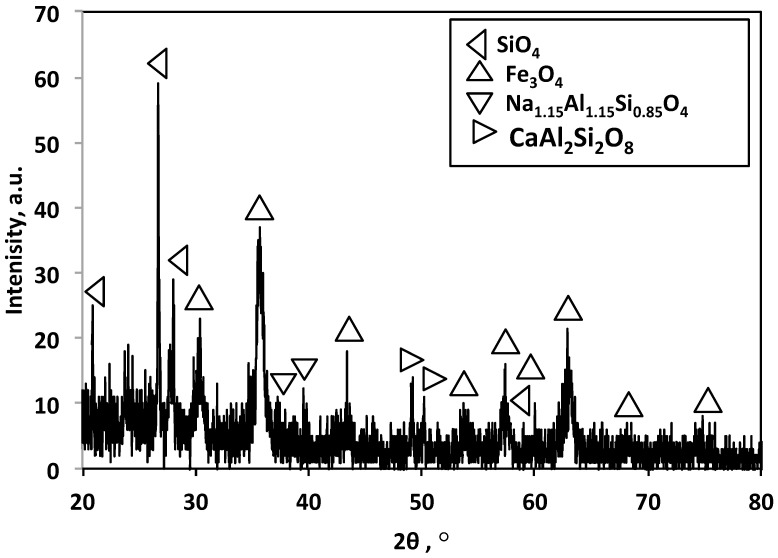
XRD pattern of the hybrid composite (magnetite/aluminum waste) material.

The crystal structure of the composite powder was explored via X-ray diffraction; the XRD results are depicted in Figure 2. The X-ray diffractogram of the prepared sample in as-synthesized form displayed the characteristics of Fe_3_O_4_, signified at 30.0, 35.5, 43.0, and 59.9°, corresponding to the peaks of [220], [311], [400], [511], and [440], respectively [22,23,24]. Furthermore, well-defined sharp diffraction peaks that signify the contribution of complex phases of sodium aluminum silicate (Na_1.15_Al_1.15_Si_0.85_O_4_) and calcium aluminum silicate (CaAl_2_Si_2_O_8_) showed a good crystalline phase. Hence, the XRD pattern proved the coexistence of Fe_3_O_4_, Na_1.15_Al_1.15_Si_0.85_O_4_, and CaAl_2_Si_2_O_8_, signifying that the composite is a multiphase system including crystalline and amorphous phases identifiable by the sharp diffraction peaks and background. Notably, the crystallinity of the Fe_3_O_4_ was retained in the substance.

#### 3.1.2. TEM Images

**Figure 3:**
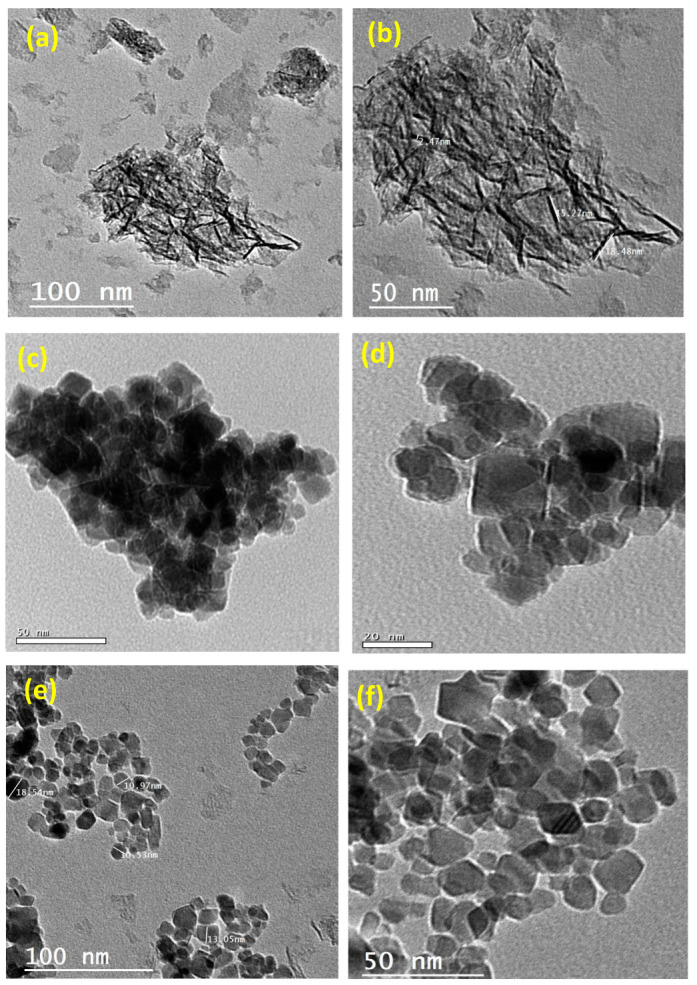
TEM images at different magnifications of (**a**,**b**) aluminum-based waste, (**c**,**d**) Fe_3_O_4_, and (**e**,**f**) composite material.

Figure 3 presents TEM images at different magnifications of the aluminum-based waste, Fe_3_O_4_, and composite material. It is obvious from the images in Figure 3a,b that the particles are of mixed shapes with predominantly varied hexagonal-like particles. Figure 3c,d shows TEM images of Fe_3_O_4_ nanoparticles, which can be categorized as a uniform spherical shape. As seen from Figure 3e,f, SEM images of the composite material revealed the presence of Fe_3_O_4_ nanoparticles, which were well deposited on the surface of the aluminum-based waste. The spherical shape of Fe_3_O_4_ is clearly apparent on the surface of the hexagonal-like particles in the composite.

#### 3.1.3. SEM Micrographs and EDX

**Figure 4:**
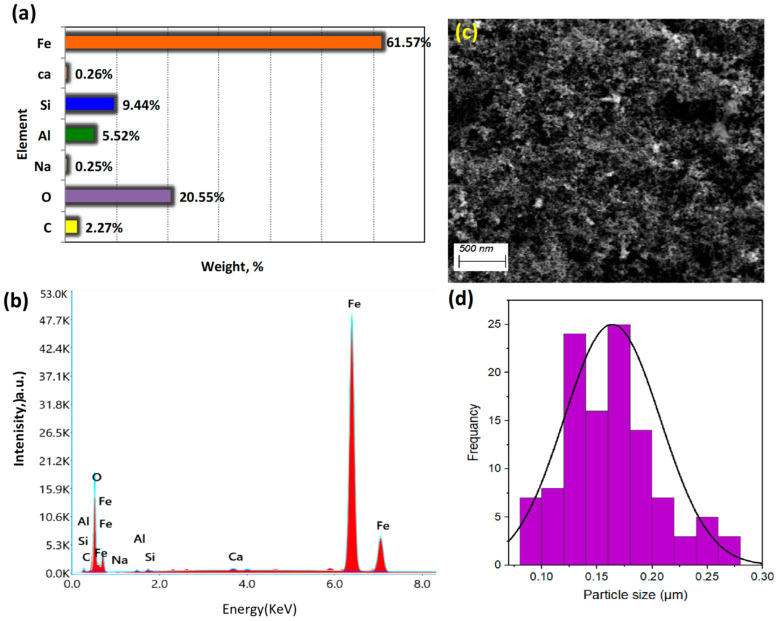
Characterization of the composite: (**a**,**b**) energy-dispersive X-ray (EDX) analysis; (**c**) SEM micrograph of the composite; (**d**) particle size distribution.

An elemental analysis of the composite sample was performed via energy-dispersive X-ray (EDX) analysis to explore the elemental content of the prepared aluminum-based waste powder augmented with magnetite nanoparticles. The results are illustrated in Figure 4a,b. The graph reveals that the composite sample comprised dominant elements of Si, Al, and Fe, suggesting it to be a successful hybrid PCM.

Additionally, Figure 4c presents SEM images that were used as an indication to explore the morphology and size of particles in the prepared composite. The SEM micrograph of the composite shows a semi-hexagonal sheet of aluminum-based waste possessing a quantity of augmented semi-spherical magnetite nanocrystals. Spherical Fe_2_O_3_ nanoparticles augmented on the spherical sheets are displayed. Moreover, the average particle size of the hybrid composite was in the micro-range, as displayed in Figure 4d. The average particle size of the incorporated magnetite nanoparticles was 42 nm, conjugated with the sheets of aluminum silicate material derived from aluminum-based waste sludge, with an average size of 0.17 µm.

### 3.2. Thermal Energy Storage Performance

#### 3.2.1. Studies on the PCM Charging/Discharging System

**Figure 5:**
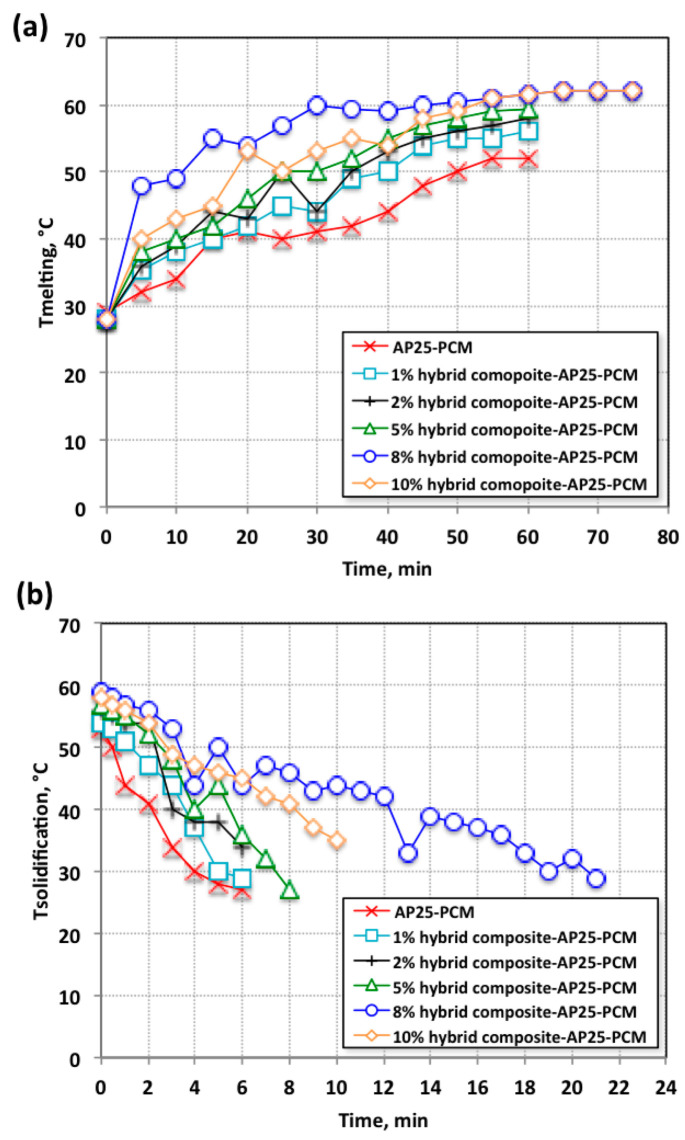
Temperature profile dissemination of the pristine AP25 and hybrid composite–AP25 PCM during the (**a**) melting cycle and (**b**) solidification cycle.

Thermal energy storage systems are assessed in this section by analyzing the data in terms of heat transfer enhancement. Charging and discharging based on melting/solidification cycles were used as an indicator for the various systems, based on the various composite concentrations. The composite contents embedded in the PCM were of varied weights of 1%, 2%, 5%, 8%, and 10%. The charging and discharging temperature profiles of the hybrid composite–wax phase change materials with different cycles for various time intervals are displayed in Figure 5. Different mass fractions were embedded in the base paraffin-AP25 wax to investigate the optimum percentage added to the PCM. Notably, as illustrated in Figure 5a, the composite fractions embedded in the paraffin-AP25 PCM revealed various ranges of melting temperatures. A temperature upsurge was observed after the addition of composite into the paraffin-AP25 at rates of up to 8%. However, further addition of composite into the paraffin-AP25 wax PCM system at rates above 8% retarded this temperature elevation.

In particular, as previously reported in the literature, composite PCMs differ from their pristine counterparts due to a convincing change in the shape of the heat flow of the wax [25,26]. Therefore, such modifications might alter the value of the melting (charging) temperature of paraffin-AP25 PCM when modified and embedded in a hybrid composite. Also, it was projected that the hybrid composite embedded in the AP25 wax would improve its latent heat. Moreover, it is noteworthy to mention that the presence of hybrid composite in the AP25 substance is governed by photodegradation activity. This could be linked to the various contents of the hybrid composite, as inferred from EDXanalysis (Figure 4a) illustrating the presence of peaks corresponding to Si, Al, and Fe [27].

The results of the solidification cycle for the pristine AP25 and the systems embedded with hybrid composite are exhibited in Figure 5b. An increase in the AP25 PCM melting temperature is clear, with a corresponding increase in the AP25 PCM solidification temperature, which was also altered according to the embedded weight of hybrid composite in the PCM. This is also associated with the upsurge in the AP25 PCM solidification time profile. These several PCM system improvements were attained via the discharging temperatures of the hybrid composite–AP25 PCM with the addition of an 8% weight fraction of hybrid composite. This might be associated with the presence of the hybrid composite dispersed in the host AP25 PCM, which could provide more inorganic sites and thereby increase the opportunity for absorbing heat, thus providing more latent heat of fusion for the paraffin-AP25 wax embedded within the hybrid composite. However, the mass fraction increase is unfavorable since it leads to a decrease in the solidification temperature profile. Such a result was previously reported and noticed in the experimental work of various researchers [28,29,30]. This might be explained by the extra addition of the embedded substance reducing the stability of the PCM, rather than enhancing its ability to store heat. This is due to the agglomeration and sedimentation that might occur, diminishing the PCM’s efficiency. Overall, selecting an optimal mass percentage of embedded material is significant for the charging and discharging system. The optimal hybrid composite PCM addition was recorded when 8-weight % was added to the pure paraffin. This might be associated with the presence of a sufficient amount for the change in shape of the heat flow of the PCM, thereby modifying the melting temperature of the PCM with additives and enhancing the latent heat of the PCM. However, extra additions to the PCM above the optimal value cause aggregates and does not further improve the PCM [15,30].

Notably, as seen in Figure 5b, the cycle time of the discharging time profile (the time taken to acquire the heat stored through the discharging cycle) of the 8% hybrid composite–AP25 PCM was increased by approximately 22 min in comparison to that for the pristine AP25 PCM. Consequently, the hybrid PCM needed a longer time than the pure PCM to finish its discharging cycle. The transient temperature profiles of the embedded PCM (hybrid composite–AP25 PCM) presented an additional picture of the melting procedure. Heat transfer occurs via conduction from the initiation of the heating cycle until the wax temperature reaches the melting temperature. Accordingly, the initial melting of the hybrid composite–AP25 PCM was produced via a complex combination of conduction and convection heat transfer. However, this was not present with the progress of the melting cycle, since the temperature increase was comparatively accelerated. This is attributed to the increase in natural convection of the melted PCM (AP25) substance [31].

#### 3.2.2. Heat Storage Density of Hybrid Composite–AP25 PCM

**Figure 6:**
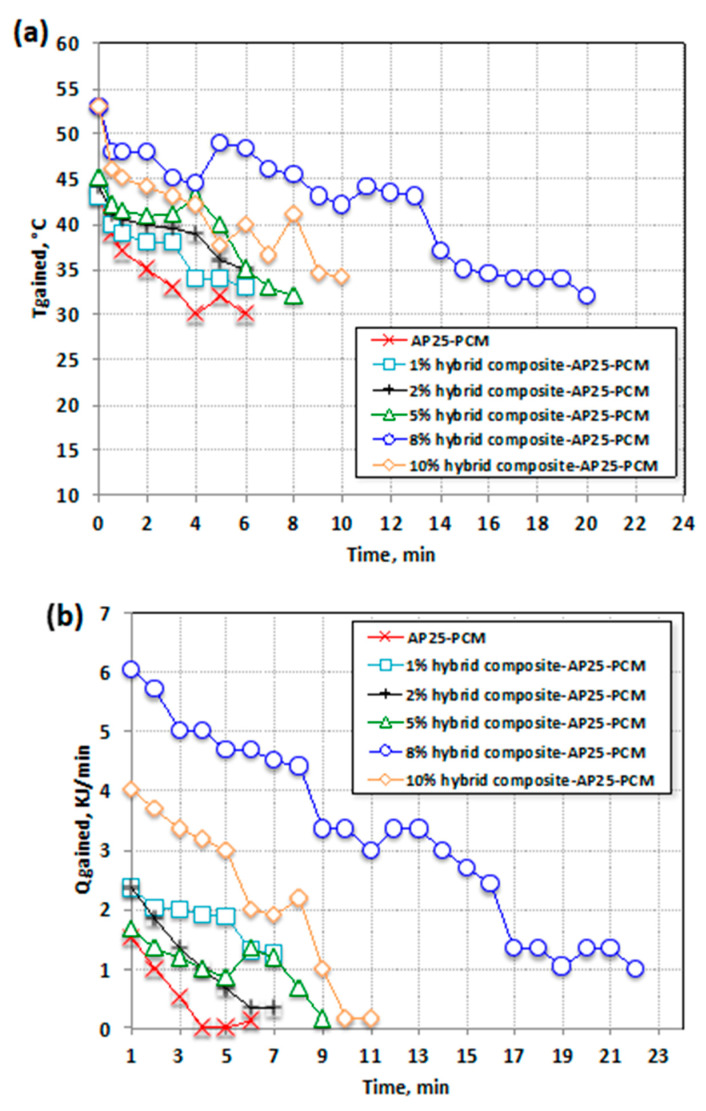
Presentation of heat storage change using various AP25-based PCM systems: (**a**) temperature profile gained from AP25 PCM; (**b**) heat flow rate during the discharging solidification cycle from AP25 PCM.

In order to extrapolate the system’s storage efficiency and capacity, the thermal reliability of the hybrid composite–AP25 PCM was determined by examining the gained temperature and heat. The data exhibited in Figure 6a,b for the AP25 wax embedded with the hybrid composite show the enhancement in the temperature that could be stored by the PCM. The incorporation of the hybrid composite in an optimal weight ratio of 8% resulted in an enhancement of the heat storage temperature to 22 °C, consequently raising the heat stored compared to that for pure AP25 PCM.

The quantity of heat obtained from the heat transfer carrier was calculated according to Equation (1) [32]:

(1) $$Q_{gained}=\dot{w}Cw\theta_{w}$$

where $\dot{w}$ is the mass flow rate of the heat transfer fluid (g/s); θ is the temperature difference of the inlet and outlet water of the heat exchanger; and C_w_ is the specific heat capacity of the heat transfer fluid.

According to the data in Figure 6b, the increase in obtained heat from the PCM systems in the initial time profile extended only to 2.3 kJ/min for the pristine-type AP25 PCM. However, the stored heat rose to 8.2 kJ/min for the hybrid composite–AP25 PCM. This could be linked to the significant role of the hybrid composite in improving the thermal transfer rate [33]. Overall, Figure 6b also shows that the pure AP25 PCM system displayed a minimal rate of heat acquired via the stored hot water in comparison to the AP25 PCM with hybrid composite. However, it is notable that the quantity of embedded filler was also a significant factor in performance. This is due to the superior thermal conductivity of the embedded metals in the PCM [13]. However, the deviation between the melting temperature and solidification temperature might be associated with the deposition of the embedded hybrid composite in the discharging cycle. The main reason for this is still unclear to the authors; further detailed experiments with various substances are required to verify such a suggestion.

In particular, the addition of inorganic material embedded in the organic paraffin PCM helps to encourage a change in the PCM’s shape during heat flow. Such a modification changes the melting temperature of the PCM conjugated with the inorganic additive. Additionally, it is expected that the additional particles incorporated in the PCM enhance its latent heat. In a comparison of the discharging cycles of the pure paraffin PCM and the composite PCM, both the melting and solidification temperature were increased. Thus, the overall heat stored from the process was increased. A significant enhancement was attained that was directly linked to the amount of composite added to the PCM. This phenomenon is usually observed experimentally, and extra composite causes agglomeration and sedimentation [33].

#### 3.2.3. Overall System Performance Efficiency

**Figure 7:**
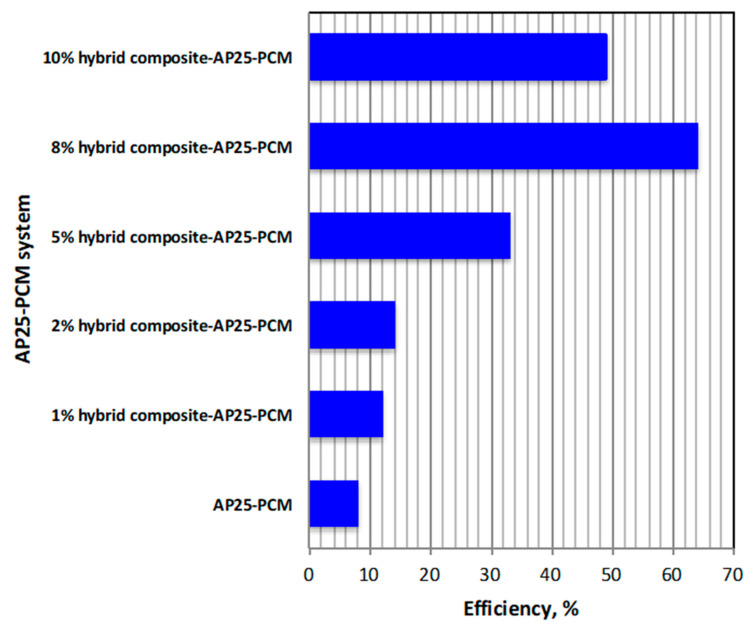
Overall system efficiency.

The overall thermal storage efficiency of the AP25 PCM and hybrid composite–AP25 PCM systems with various embedded mass fractions was examined and compared, as presented in Figure 7. The heat gained from the heat transfer carrier, given by Equation (1), was required to calculate the overall efficiency.

Additionally, the heat gained from the AP25 PCM substance was calculated via Equation (2) to obtain the system efficiency:

(2) $$Q_{PA25-\mathrm{PCM}\;\mathrm{system}}=m_{\mathrm{PCM}}C_{\mathrm{PCM}}\theta_{PCM}+m\;L_{f}$$

where $m_{PCM}m_{PCM}$ is the mass of PCM (Kg), $C_{PCM}C_{PCM}$ is the specific heat capacity of the PCM (kJ/kg·K), and $\theta_{PCM}\;\theta_{PCM}$ corresponds to the T_PCM_, which is the difference in temperature going into and out of the heat exchanger. Thus, the system efficiency was calculated using Equation (3), by dividing the heat gained (Equation (1)) by the heat gained from the AP25 PCM (Equation (2)).

(3) $$Efficiency(\%)=\frac{Q_{gained}}{Q_{{PA25-\mathrm{PCM}\;\mathrm{system}}_{}}}\times 100$$

The results are displayed in Figure 7. It is clear from the figure that the efficiency improved by 64% with the inclusion of 8% embedded hybrid composite, which is in agreement with the abovementioned results.

#### 3.2.4. Comparison of Various PCM Systems

Numerous PCM systems improved via the use of various additives, and those reported on in several studies in the literature were compared with that in the current study; the data are tabulated in Table 2. This comparison is based on the efficiency enhancement. A reasonable enhancement was achieved via the addition of inorganic materials. The efficiency of the PCM in the current investigation was among the highest values. Overall, the use of inorganic fillers allows excellent thermal performance compared to the pristine PCM. Although a greater improvement in efficiency was reported in other studies [13,28,34,35,36,37,38,39,40,41], it is notable that the current investigation is based on the use of a waste stream, which is in line with the trends of industrial ecology and cost-effectiveness.

The most common advantages of inorganic PCMs are their high enthalpy and conductivity, low cost, high storage capacity, and high temperature storage range. However, corrosive substances or those with low specific heat could display segregation and a lack of stability. Such demerits of inorganic PCMs have motivated scientists to search for a reasonable solution to improve their efficiency [36]. These results testify to the hypothesis that the presence of inorganic added material in a PCM confers greater heat storage than that achieved by pure paraffin PCM, along with a higher discharging temperature [42].

## 4. Conclusions

Low thermal conductivity remains the main obstacle to the commercialization of thermal energy storage using phase change materials, in addition tithe toxic and corrosive properties of some PCMs. The results obtained in the current study showed that the thermal conductivity of paraffin-AP25 was increased by 64% when 8 wt% composite was added into the paraffin-AP25. The composite notably enhanced the thermal conductivity of the paraffin-AP25. Additionally, the amount of heat stored was elevated by the addition of 8% composite, which corresponded to the best performance. Such PCM systems are good examples of systems that might be approved as a simple and cost-effective alternative to avoid the use of expensive substances in energy storage facilities. However, further work is essential to evaluate the thermal cycle layouts of the hybrid composite–AP25 PCM and to determine the reusability of the suggested material.

## Figures and Tables

**Table 1:** Thermophysical characterization of paraffin-AP25 wax.

Property	Value	Unit
Melting temperature	48–53	°C
Latent heat of fusion	190	kJ/kg
Solid density	930	kg/m^3^
Liquid density	830	kg/m^3^
Thermal conductivity	0.21	kJ/kg°C
Solid specific	2.1	kJ/kg°C
Density (solid/liquid)	833/775	kg/m^3^
Kinematic viscosity	8.3 × 10^−5^	m^2^/s

**Table 2:** A comparison of the PCM investigated in thecurrent studywith those from other studies in the literature, in terms of efficiency.

Composite PCM Type	Efficiency Enhancement	Ref.
Hybrid Fe/Al/Si	64%	Current study
Zeolite	85%	[34]
Graphene nanoplates	49%	[35]
SiO_2_	6%	[36]
Cu	52%	[37]
Si_3_N_4_	77.4%	[38]
ZnO/SiO_2_	19%	[39]
Graphene nanoplatelets	100%	[40]
Carbon nanotubes	86%	[41]
Aluminum nitride	36.47%	[28]
TiO_2_	0.46%	[13]

## Data Availability

Data are available upon request.
